# Long-Term Potentiation-Like Visual Synaptic Plasticity Is Negatively Associated With Self-Reported Symptoms of Depression and Stress in Healthy Adults

**DOI:** 10.3389/fnhum.2022.867675

**Published:** 2022-04-22

**Authors:** Trine Waage Rygvold, Christoffer Hatlestad-Hall, Torbjørn Elvsåshagen, Torgeir Moberget, Stein Andersson

**Affiliations:** ^1^Department of Psychology, Faculty of Social Sciences, University of Oslo, Oslo, Norway; ^2^Department of Neurology, Oslo University Hospital, Oslo, Norway

**Keywords:** LTP (long term potentiation), visual evoked potential (VEP), EEG, stress, depressive symptoms

## Abstract

Long-term potentiation (LTP) is one of the most extensively studied forms of neuroplasticity and is considered the strongest candidate mechanism for memory and learning. The use of event-related potentials and sensory stimulation paradigms has allowed for the translation from animal studies to non-invasive studies of LTP-like synaptic plasticity in humans. Accumulating evidence suggests that synaptic plasticity as measured by stimulus-specific response modulation is reduced in neuropsychiatric disorders such as major depressive disorder (MDD), bipolar disorders and schizophrenia, suggesting that impaired synaptic plasticity plays a part in the underlying pathophysiology of these disorders. This is in line with the neuroplasticity hypothesis of depression, which postulate that deficits in neuroplasticity might be a common pathway underlying depressive disorders. The current study aims to replicate and confirm earlier reports that visual stimulus-specific response modulation is a viable probe into LTP-like synaptic plasticity in a large sample of healthy adults (*n* = 111). Further, this study explores whether impairments in LTP-like synaptic plasticity is associated with self-reported subclinical depressive symptoms and stress in a healthy population. Consistent with prior research, the current study replicated and confirmed reports demonstrating significant modulation of visual evoked potentials (VEP) following visual high-frequency stimulation. Current results further indicate that reduced LTP-like synaptic plasticity is associated with higher levels of self-reported symptoms of depression and perceived stress. This indicate that LTP-like plasticity is sensitive to sub-clinical levels of psychological distress, and might represent a vulnerability marker for the development of depressive symptoms.

## Introduction

### Background

Neuroplasticity represents a fundamental property of the human brain that enables us to both sense, assess and store complex information and subsequently make appropriate, adaptive responses to related stimuli (Liu et al., 2017). More specifically, synaptic plasticity refers to the activity-dependent modification of synaptic transmission (Citri and Malenka, 2008). Long-term potentiation (LTP) is a phenomenon which entails the enhancement of synaptic transmission through simultaneous pre- and post-synaptic activity leading to long-lasting modification of synaptic strength. Originally demonstrated *in vivo* in the rodent hippocampus (Bliss and Lomo, 1973), LTP has since been extensively investigated and has been demonstrated to extend to areas outside of the hippocampus such as the visual cortex (Sanders et al., 2018). LTP involves an increase of excitatory synaptic transmission potentially lasting hours to weeks, and is regarded as the most promising candidate synaptic model for describing the basic cellular mechanisms underlying memory and learning (Jahshan et al., 2017).

Whereas LTP has been widely studied in animals and *in vitro* hippocampal slices, there is a paucity of evidence regarding synaptic plasticity in humans due to the lack of non-invasive methods for assessing the phenomenon *in vivo*. In recent years, however, studies using sensory event-related potentials (i.e., visual evoked potentials; VEP) as a proxy have approached the study of synaptic plasticity in the intact human brain (Clapp et al., 2005; Teyler et al., 2005). Specifically, modification of VEP amplitudes following repeated visual stimulation have shown considerable promise as a non-invasive *in vivo* assay of synaptic plasticity and function (e.g., Teyler et al., 2005). In these studies, experimental paradigms typically record a VEP baseline prior to introducing high-frequent or prolonged visual stimulation to induce modulation of visually evoked potentials post-stimulation (e.g., Spriggs et al., 2019; Valstad et al., 2020; Lengali et al., 2021; Rygvold et al., 2021). These experimental VEP paradigms are typically designed to be analogous to invasive procedures inducing synaptic plasticity in brain slices (Normann et al., 2007). This stimulus-specific response modulation resembles LTP induced by electrical stimulation in animal studies, and shares several core defining characteristics of LTP, such as longevity, input specificity and *N*-methyl-D-aspartate (NMDA)-receptor dependency (Kirk et al., 2010).

### Synaptic Plasticity, Stress, and Depression

Stress is generally regarded as an important precipitating factor in the development of psychiatric disorders, including depressive disorders (Liu et al., 2017). Both stress and depression are associated with biomarkers such as elevated cortisol levels and hypothalamic-pituitary-adrenal (HPA) axis hyperactivity, and both elevated stress levels and depressive symptoms have a negative impact on cognitive functions such as learning and memory (Duman et al., 2017). Though mild transient stress can be adaptive and facilitate memory and learning processes, severe or chronic stress have detrimental effects on the central nervous system (Pittenger and Duman, 2008). The effect of stress on the human central nervous system thus seems to coincide with the Yerkes–Dodson’s law, stating that the association between stress and performance follows an inverted U-curve (Diamond et al., 2007). Impairments in hippocampal-dependent explicit memory functions have been demonstrated in both animal and human subjects after treatment with glucocorticoids (reviewed in Shors, 2006). Animal research have demonstrated that chronic exposure to glucocorticoids can lead to atrophy of hippocampal neurons, similar to what is seen in the human hippocampus in depression and other neuropsychiatric conditions. Furthermore, stress also impairs the expression of LTP and even facilitates long-term depression (LTD) through increased glucocorticoid exposure, as shown in animal models of depression (Player et al., 2013).

In recent years, impaired LTP-like synaptic plasticity, indexed as reduced modulation of VEP amplitudes following visual stimulation, has been implicated in the pathophysiology of a variety of psychiatric disorders such as major depressive disorder (MDD) (Normann et al., 2007), bipolar II disorder (Elvsåshagen et al., 2012; Zak et al., 2018), and schizophrenia (Çavuş et al., 2012; Jahshan et al., 2017; Valstad et al., 2021). Additionally, studies have reported a relationship between symptom severity and magnitude of the plasticity impairment, including significant associations between VEP amplitude modulation and clinical rating scales measuring depression, indicating larger impairment in synaptic plasticity in the more severely depressed individuals (Elvsåshagen et al., 2012; Zak et al., 2018). Recent research have also argued that the impairment in synaptic plasticity is state-dependent, and show a restoration in remission from depressive disorders (Kuhn et al., 2016). Seen together, these results lend support to the neuroplasticity hypothesis of depression, stating that impairments in neuroplasticity might represent a common pathway underlying depressive disorders (Liu et al., 2017).

Current integrative models of neuroplasticity and depression implicate exposure to chronic or traumatic stress in inducing both deficits in hippocampal neurogenesis as well as synaptic connectivity in the hippocampus and the prefrontal cortex (PFC), consequently leading to impairments in cognitive functions such as memory and learning (Price and Duman, 2020). Research on the effects of antidepressants lends some support to the neuroplasticity hypothesis of depression, indicating that the antidepressant effect of these psychotropics is a result of inducing a plasticity effect that in turn facilitate greater benefits of psychotherapy (Castrén and Hen, 2013). This indicates a possible synergetic effect between antidepressants’ effect on mood state and their possible enhancement of adaptability in the central nervous system through mechanisms of synaptic plasticity. Normann et al. (2007) found that both baseline and post-modulation VEP amplitudes increased in healthy control subjects when they were treated with a selective serotonin reuptake inhibitor (SSRI) over a time period of 3 weeks, which supports this assumption (Normann et al., 2007). Conversely, chronic stress will reduce this structural variability (Castreń, 2013). Further support for plasticity mechanisms in depressive states comes from research on the glutamatergic agent ketamine, which exhibits well-replicated rapid-acting anti-depressant effects in randomized, controlled trials of major depression (Xu et al., 2016). There is also some evidence that the administration of ketamine might have an antidepressant effect in conditions that are difficult to treat, such as bipolar depression (Dean et al., 2021) and treatment-resistant depression (Caddy et al., 2015). The antidepressant efficacy of ketamine has been attributed to its neuroplastic abilities, including increasing brain-derived neurotrophic factor (BDNF) and formation of new synapses in animal models (Price and Duman, 2020). The dynamics surrounding neuroplasticity, stress and depression needs to be further elucidated, especially considering the heterogeneous nature of depressive disorders. However, there seem to exist a distinct association between the effects of stress, the mechanisms of synaptic plasticity and the pathophysiology of depression.

Both level of perceived stress and affective symptoms in an individual exist on a continuum from asymptomatic to severe symptoms, and though there are clinical guidelines concerning cut-off points on self-report measures that indicate clinically relevant depressive symptomatology (e.g., Beck et al., 1996), what constitutes relevant subclinical depressive symptoms is not as clearly defined. In addition, self-reported symptoms of stress, affective symptoms and executive function tend to correlate highly, as they reflect a general experience of psychological distress. Subclinical depression is conceptualized as part of the prodromal phase of depression, and is one of the most robust predictors of major depression (Van Zoonen et al., 2015). With this in mind, it might be plausible to expect changes in synaptic plasticity even in individuals with subclinical symptoms of depression.

We have previously, in line with several other studies of VEP-modulation, demonstrated robust modulation of VEP amplitudes using high-frequency visual stimulation in healthy participants (Rygvold et al., 2021). The current study is an extended analysis based on this earlier work, with a somewhat higher *n* (101 vs. 111). The main aim of this study is to explore the association between impaired synaptic plasticity and perceived level of stress, self-reported depressive symptoms, and self-reported executive function in a healthy population. To our knowledge, no previous studies have explored these associations linking subclinical level of depression, stress, and executive dysfunction to LTP-like synaptic plasticity in healthy adults. In addition to replicating previous reports on modulation of VEP amplitudes after high-frequency stimulation, we expect to observe a negative association between LTP-like synaptic plasticity and self-reported stress and depressive symptoms. This potential association might indicate that impaired LTP-like synaptic plasticity represents an underlying vulnerability marker for the development of clinical depressive symptoms.

## Materials and Methods

### Participants

Using the G*Power 3.1.9.7. software (Erdfelder et al., 2009) a *post-hoc* power estimate of 1-β = 1.00 was calculated for a repeated measure, within-factors ANOVA (6 levels, α = 0.05; *f* = 0.25) to assess possible modulation effects of the visual high-frequent stimuli. A *post-hoc* power estimate of 1-β = 0.89 was calculated for a two-tailed bivariate correlation with a medium effect size of 0.30 and *n* = 111 (α = 0.05) to assess possible associations between LTP-like synaptic plasticity and self-reported depressive symptoms and stress.

A total of 111 healthy subjects provided informed consent and participated in the study (69 females, 42 males; mean age 37.6 years, SD 13.97, range 17–71). Self-reported normal or corrected-to-normal vision, no ongoing substance abuse or use of psychoactive medication, and absence of any current or previous severe psychiatric or neurological condition was required. Participants were recruited through social media platforms (Facebook, Instagram), in addition to local advertisement. The regional ethics committee for medical research approved all procedures (ref. no: 2016/2003).

### Data Acquisition

Electroencephalographic (EEG) data were recorded using a 64-channel (Ag-AgCl electrodes) BioSemi ActiveTwo system (BioSemi B.V., Amsterdam). The electrodes were spatially positioned according to the international, extended 10–20 system (10-5; Oostenveld and Praamstra, 2001). Four additional external electrodes were positioned around the eyes; laterally, and inferior/superior to the right eye (corresponding to the 10–20 system locations of LO1, LO2, IO2, and SO2); and at each earlobe (locations A1 and A2). Raw data was recorded at a sampling rate of 1,024 Hz. No online filters were applied, only a hardware anti-aliasing filter was used. A 25-pin serial port was used to send event markers from the MATLAB platform to the EEG acquisition software.

### Experimental Setup

**FIGURE 1:**
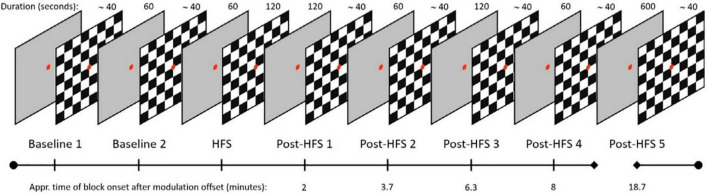
Layout of the VEP paradigm, boxes indicating duration of blocks in seconds, time of block onset after HFS displayed on timeline.

The experimental protocol to demonstrate LTP-like modulation of sensory evoked potentials consisted of one visual and one auditory paradigm, run sequentially. In addition, a period of resting-state EEG recording and a loudness dependence of the auditory evoked potential (LDAEP) paradigm. The session lasted approximately 50 min (see Figure 1 for the protocol layout). Previous research from our group have reported more modest modulation effects of the auditory evoked potential paradigm (AEP), as well as reporting no significant within-subject correlations between modulation in the two sensory modalities (Rygvold et al., 2021). For these reasons, the current paper will report results from the visual stimulus-selective response modulation (SRM) paradigm only.

Participants were comfortably seated 70 cm from a 24” LCD screen (BenQ, model ID: XL2420-B) on which visual stimuli were presented. The visual stimuli was programmed in the Psychtoolbox-3 environment (Kleiner et al., 2007) and run on the MATLAB platform (version 2015a; MathWorks, Natick, MA, United States). For the 120 s high frequency stimulation (HFS) block, the stimulus reversal frequency was locked to ∼8.55 Hz, customized to fit the monitor refresh frequency at 60 Hz. The participants were given verbal instructions prior to the experimental session, as well as written paradigm-specific reminders on-screen before the onset of each paradigm. When not reading instructions, participants were required to fixate on a red circular dot centrally positioned on the screen.

The visual SRM paradigm consists of two baseline VEP blocks, one HFS block, and five post-HFS blocks which were identical to the baseline blocks. Each pre-/post-HFS block consisted of 40 trials, including five target trials. One *trial* corresponds to one reversal of a black and white checkerboard texture (check size approximately 1.0°), whereas *target* trials refer to trials cueing the participants to press a response button, by switching the color of the fixation dot briefly to green. The target trials were included to ensure attention and prevent fatigue and drowsiness during the experimental session but were not included in the final VEP analyses. Trials in the pre-/post HFS blocks were separated by a random stimulus onset asynchrony (SOA) value in the 500–1500 milliseconds range (mean reversal rate; 1 Hz). In the HFS block, the stimulus reversal frequency was locked to ∼8.55 Hz, corresponding to a SOA of ∼0.143 s. Each pre- and post HFS block had a duration of approximately 40 s, the HFS block was 120 s. The post-HFS blocks were recorded approximately 2, 4, 6, 8, and 19 min after the baseline block, respectively.

Visual evoked potentials components C1, P1, and N1 were identified individually for each subject by visual inspection. This was done by defining a time window for all peaks from the grand average for each participant (C1: 80–105 ms, P1:110–145 ms, N1: 150–250 ms), and subsequently marking the amplitude peaks manually if they deviated in latency from the pre-set temporal windows. Subsequently, block-specific peaks were defined as the minimum/maximum amplitude data points inside this time window. Amplitude was recorded at these subject-specific latencies separately for each block. In addition, the P1-N1 peak-to-peak amplitude was computed. The measurements were obtained from an occipital electrode cluster (mean amplitude of O1, Oz, and O2). This electrode cluster was selected in order to capture the maximum difference between baseline and post-HFS blocks. The two pre-modulation blocks were averaged into one block, labeled Baseline (BL). Post-HFS blocks were analyzed separately and labeled Post-HFS 1, 2, 3, 4, and 5, respectively.

### Electroencephalographic Pre-processing

The EEG data were preprocessed in the EEGLAB (version 2021.1) environment (Delorme and Makeig, 2004) on the MATLAB platform (version 2019b). Continuous EEG data were re-referenced to the average of the 64 EEG channels and resampled to half of the original sampling rate. EEG segments containing visual SRM data was extracted, and data with no relevance to the visual paradigm was discarded from further pre-processing.

A lower bound 1 Hz high-pass filter (EEGLAB default, with data edge padding) was applied to remove the DC offset as well as low-frequency drifts. Channels with an amplitude SD outside an interval of 1–25 μV were removed from the reference signal iteratively. The ZapLine tool (de Cheveigné, 2020) and an upper bound 30 Hz low-pass filter (EEGLAB default, with data edge padding) were used to suppress line noise and high-frequency noise, respectively. Portions of the data which displayed significant noise in >50% of the channels were rejected. In addition, the remaining channels with excessive noise in >10% of the remaining data points were removed. Signal artifacts attributable to eye blinks and facial movements were removed using independent component analysis (Delorme and Makeig, 2004). The EEGLAB implementation of the second-order blind separation algorithm (Belouchrani et al., 1993) was used for component decomposition. Ocular components were identified with the ICLabel tool (Pion-Tonachini et al., 2019). A final removal of noisy channels was carried out with tools from the PREP pipeline toolbox (Bigdely-Shamlo et al., 2015). All of the removed channels were then spherically interpolated. Visual SRM epochs were extracted into separate files from the cleaned data. Epochs were time-locked to the onset of the visual stimuli with an epoch length of 500 ms, including a pre-stimulus period of 100 ms for baseline correction. Epochs containing signals above/below ± 50 μV in channels spatially relevant to the subsequent ERP measurement were rejected. The signals were re-referenced to AFz prior to peak measurements.

### Self-Report Questionnaires

Self-reported depressive symptoms at the time of the VEP recording were assessed with Beck Depression Index-II (Beck et al., 1996), symptoms of stress were assessed with the Perceived Stress Scale-10 (Cohen et al., 1994) and self-reported executive function was assessed with Behavior Rating Inventory of Executive Function-Adult (Roth et al., 2005).

The BDI-II is a 21-item self-report measure of depressive symptoms (experienced during the last 2 weeks) that is widely used in both clinical and non-clinical populations. When employing the BDI-II in large samples of healthy controls, the distribution will typically be skewed with a mean score in the area 4 to 6 (Kendall et al., 1987), and the mean in the current population is 4.55. With this in mind, a cut-off point of = > 10 is suggested to distinguish the non-depressed from individuals with some degree of depressive symptoms closely related to dysphoria or clinically relevant mild depression (Kendall et al., 1987; Koster et al., 2005). A two-factor structure of the BDI-II has been proposed, one factor loading onto items pertaining to the somatic aspects of depressive symptomatology (e.g., *changes in appetite* and *changes in sleeping patterns*) and the other factor loading onto items intended to measure the cognitive-affective aspects of depression (e.g., *self-criticalness* and *loss of interest*) (Beck et al., 1996; Whisman et al., 2003). The possible unique contribution of these two factors on the degree of SRM-plasticity will be considered. Questionnaire data from one participant is missing. In addition, five participants did not complete page three of the BDI-II, so that the score on the two latent factors was not calculated. A total BDI-II score was, however, calculated for these five participants based on the available questionnaire data (15/21 items).

The Perceived Stress Scale-10 (PSS-10) is a self-report measure intended to measure the degree of perceived stress relative to the ability to cope with stressful situations experienced during the last month (Cohen et al., 1994; Taylor, 2015). The PSS is not designed to be a diagnostic tool but may aid clinicians in identifying prodromal stages of psychiatric disorders (Cohen et al., 1994). Several studies have identified two latent factors in the PSS-10, with negatively phrased items loading on a “perceived helplessness” scale (e.g., *In the last month, how often have you felt that you were unable to control the important things in your life?*) and positively phrased items loading on a “perceived self-efficacy” scale (e.g., *In the last month, how often have you felt that things were going your way?*) (e.g., Storch et al., 2004). Both factors have been shown to predict depression in women, whereas only the perceived helplessness scale have been shown to predict depression in both men and women (Taylor, 2015). Questionnaire data from one participant was missing. An additional participant did not complete all items in the questionnaire, so that only the total score and the score on the subscale “perceived helplessness” could be calculated.

The Behavioral Rating Inventory of Executive Function-Adult version (BRIEF-A) is a 75-item self-report questionnaire originally intended to measure cognitive and behavioral-affective aspects of executive dysfunction affecting daily living (Roth et al., 2005). Participants answer the following question: “*During the past 6 months, how often has each of the following behaviors been a problem?*” with responses scored as never = 1; sometimes = 2; or often = 3. The BRIEF-A produces a composite index score, Global Executive Composite (GEC), and two sub-index scores; Behavioral Regulation Index (BRI) and Metacognition Index (MI), based on nine subscales. Raw scores are transformed into age-corrected t scores. Several studies have indicated that high scores on the BRIEF-A correlate more strongly with self-reported affective symptoms than impairments in executive functions as measured by performance-based neuropsychological assessment. This is true for both individuals with mild to moderate depression (Hagen et al., 2021), as well as individuals with a variety of neurological disorders and neuropsychiatric disorders (Løvstad et al., 2016). Cut-off scores indicating clinical significance will be based on the mean scores of healthy Norwegian controls in Løvstad et al. (2016) and defined as 1.5 standard deviations above this mean.

### Statistical Analyses

To assess the main effect of the HFS block on the VEP component amplitudes, each component was tested using repeated measures ANOVAs with block (BL, post-HFS 1, post-HFS 2, post-HFS 3, post-HFS 4, post-HFS 5) as the within-subject factor. Each component was subjected to *post-hoc* paired samples *t*-tests comparing each post-HFS block VEP amplitude to the associated BL block amplitude separately. To examine the modulation effect, the difference between each post-modulation block and BL was computed by subtracting the BL score from the post-HFS scores for all components for each participant separately. Average modulation scores across blocks were computed for all components; C1, P1, N1, and P1-N1 peak-to-peak. The distribution of scores on both the BDI-II, PSS-10, and GEC score from the BRIEF-A were not normally distributed (Kolmogorov-Smirnov test of normality were all *p* < 0.05). Consequently, analyses of associations between VEP-plasticity and self-reported depressive symptoms, perceived stress and executive function were assessed using non-parametric rank-correlation analyses (Spearman’s *rho*). To assess associations between VEP plasticity and self-reported depressive symptoms, modulation scores were correlated with both the total score, as well as the somatic and cognitive-affective factors of the BDI-II. Possible associations between VEP plasticity and self-reported stress were assessed by correlating modulation scores with both the total score of PSS-10 as well as the factors measuring perceived helplessness and perceived self-efficacy. Potential associations between VEP plasticity and self-reported symptoms of executive dysfunction were assessed by correlating modulation scores with the GEC, the BRI and the MI of the BRIEF-A. To further explore a possible association between self-reported psychological distress and VEP plasticity, non-parametric two independent samples Mann-Whitney *U*-tests were employed to assess whether there were significant differences between the high- (> = 10) and low scorers on BDI on VEP plasticity indexed by average modulation scores on all components. In addition, Mann–Whitney *U*-tests were used to assess possible significant differences between individuals showing a high degree of modulation (*n* = ), defined as one standard deviation above the mean on the composite modulation scores on all components, vs. participants showing a lower degree of modulation (<one SD above the mean) on self-reported psychological distress. For all statistical analyses, a two-tailed *p*-value of < 0.05 was considered significant. Greenhouse-Geisser corrections were applied to all analyses of variance with repeated measures. Both Spearman’s *r* correlations and two independent samples *t*-tests were corrected for multiple comparisons using Bonferroni correction. Effect sizes are written as Eta-squared (ηp^2^). All statistical analyses were performed using IBM SPSS Statistics (version 27).

## Results

### Visual High Frequency Stimulation Modulation

#### Visual High Frequency Stimulation Induces Modulation Effects in All Visual Evoked Potentials Components

**FIGURE 2:**
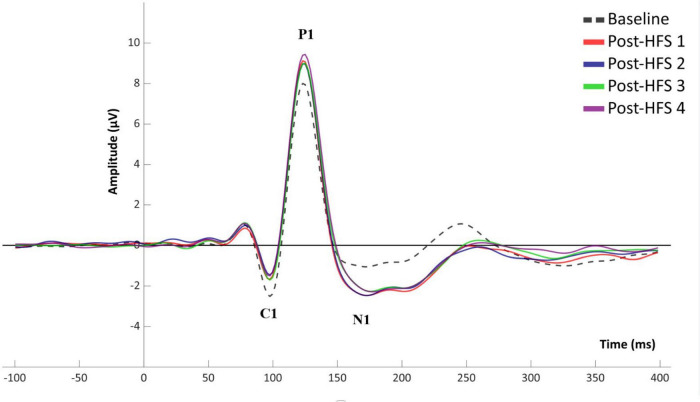
Grand average (*n* = 110) VEP waveform for baseline and post-HFS blocks.

**FIGURE 3:**
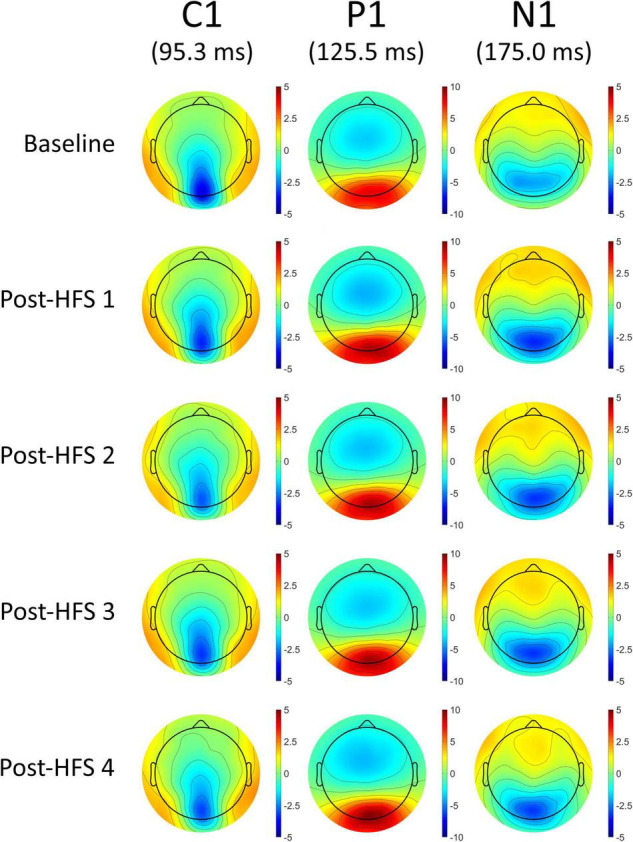
Topographical voltage maps of the VEP components C1, P1, and N1.

A significant main effect of *HFS* on amplitudes was observed in all VEP components; C1 [*F*_(4_,_522)_ = 5.845, *p* < 0.001, ηp^2^ = 0.051], P1 [*F*_(4_,_375)_ = 15.315, *p* < 0.001, ηp^2^ = 0.123], N1 [*F*_(4_,_652)_ = 12.613, *p* < 0.001, ηp^2^ = 0.104], and P1-N1 [*F*_(3_,_790)_ = 38.981, *p* < 0.001, ηp^2^ = 0.263]. See Figures 2, 3 for grand average VEP waveforms and topographical maps, respectively. As the P1-N1 component displayed the strongest modulation effects this component will be the basis of further analysis, though significant results related to other components will also be reported.

#### Block-Wise Effect of Visual High Frequency Stimulation

**FIGURE 4:**
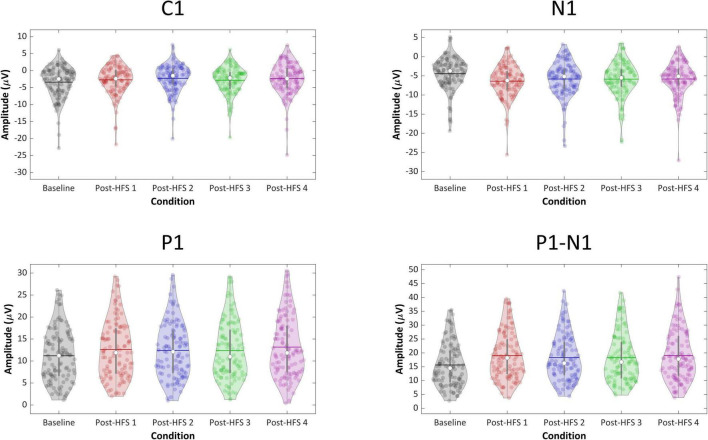
Violin plots of component VEP amplitudes (C1, P1, N1, P1-N1) across blocks.

For all VEP components, each post-HFS block was separately compared to the associated BL block (Figure 4). For the C1 component, both Post-HFS 1 [*t*(109) = −2.976, *p* < 0.004], Post-HFS 2 [*t*(109) = −5.207, *p* < 0.001], Post-HFS 3 [*t*(109) = −2.286, *p* = 0.024] and Post-HFS 4 [*t*(109) = −3.834, *p* < 0.001] displayed reduced amplitudes compared to baseline, whereas Post-HFS 5 did not [*t*(109) = −1.014, *p* = 0.313]. Similarly, for the P1 component, significant increases in amplitude from BL were observed in both Post-HFS 1 [*t*(109) = −5.749, *p* < 0.001], Post-HFS 2 [*t*(109) = −4.780, *p* < 0.001], Post-HFS 3 [*t*(109) = −3.815, *p* = 0.001] and Post-HFS 4 [*t*(109) = −6.049, *p* < 0.001] but not in Post-HFS 5 [*t*(109) = 0.815, *p* = 0.417]. The N1 amplitude showed a significant increase compared to BL in Post-HFS 1 [*t*(109) = 7.758, *p* < 0.001], Post-HFS 2 [*t*(109) = 5.429, *p* < 0.001], Post-HFS 3 [*t*(109) = 5.167, *p* < 0.001], Post-HFS 4 [*t*(109) = 4.384, *p* = < 0.001], and Post-HFS 5[*t*(109) = 2.959, *p* = 0.004]. The P1-N1 peak-to-peak component showed significantly increased amplitude compared to BL in Post-HFS 1 [*t*(109) = −12.738, *p* < 0.001], Post-HFS 2 [*t*(109) = −8.517, *p* < 0.001], Post-HFS 3[*t*(109) = −8.807, *p* < 0.001] and Post-HFS 4 [*t*(109) = −8.713, *p* < 0.001], but not Post-HFS 5 [*t*(109) = −1.690, *p* = 0.094]. All significant results survived correction for multiple comparisons (Bonferroni corrected α = 0.01) except the C1 component post-HFS 3 block. As the post-HFS 5 block yielded no modulation effects, an average of block 1–4 will be employed for further analyses. One participant only had VEP-amplitudes from post-stimulation block 5 after EEG pre-processing. Thus, this participant was excluded from further analyses.

### Associations Between Visual Evoked Potentials-Plasticity and Self-Reported Psychological Distress

#### Correlations Between Self-Report Measures of Depressive Symptoms, Stress and Executive Function

The BDI-II, PSS-10, and BRIEF-A all showed high internal consistency, with Cronbach’s α = 0.886; 0.834; and 0.902, respectively. Correlation analyses using Spearman’s *rho* showed moderate significant correlations between the total scores of BDI-II and PSS-10 [*r*_*s*_(109) = 0.442, *p* < 0.001], total score of BDI-II and the GEC score of BRIEF-A [*r*_*s*_(109) = 0.580, *p* < 0.001], and total score PSS-10 and GEC of BRIEF-A [*r*_*s*_(109) = 0.470, *p* < 0.001]. Furthermore, a moderate correlation was found between the two latent factors of the BDI-II [*r*_*s*_(104) = 0.560, *p* < 0.001], and between the two latent factors of the PSS-10 [*r*_*s*_ (109) = 0.505, *p* < 0.001].

#### Associations Between Visual Evoked Potentials Modulation and Self-Reported Depressive Symptoms

Modest significant negative correlations using Spearman’s *rho* were found between averaged P1-N1 peak-to-peak amplitude change and the total score of BDI-II [*r*_*s*_(109) = −0.319, *p* = 0.001]. Significant negative correlations were also found between P1-N1 VEP-modulation and the two factors of the BDI-II; the somatic factor [*r*_*s*_(104) = −0.299, *p* = 0.002] and the cognitive-affective factor [*r*_*s*_(104) = −0.340, *p* < 0.001]. In addition, a significant correlation was found between average N1 amplitude change and the somatic factor of the BDI [*r*_*s*_(104) = −0.245, *p* < 0.012]. All correlations survived correction for multiple comparisons (*Bonferroni corrected* α = 0.0125). Further, there were significant correlations between average modulation of the P1 component and both total score [*r*_*s*_(109) = −0.200, *p* < 0.037] and the cognitive-affective factor on the BDI [*r*_*s*_(104) = −0.233, *p* < 0.017]. A significant correlation was also found between averaged C1 modulation and both total score [*r*_*s*_(109) = 0.200, *p* < 0.037] and the somatic factor of the BDI [*r*_*s*_(104) = 0.236, *p* < 0.016]. The correlations between P1 and C1 modulation and self-reported depressive symptoms did not, however, survive correction for multiple comparisons.

A two independent samples Mann-Whitney *U*-test was conducted to compare VEP modulation in participants with high (*n* = 17) vs. low (*n* = 92) total scores on the BDI-II (described in see section “Self-Report Questionnaires”). There was a significant difference in P1-N1 modulation between the high and low BDI-II scoring group (*U* = 501, *z* = −2.394, *p* = 0.017, ηp^2^ = 0.052). There were no significant differences between the high- and low-scoring groups on any of the other VEP components; C1, N1, and P1.

A Mann-Whitney *U*-test was employed to compare self-reported depressive symptoms in participants showing higher (*n* = 16) vs. lower (*n* = 94) degree of VEP modulation. There were significant differences between averaged P1-N1 modulation on both the total score (*U* = 503.5, *z* = −2.082, *p* = 0.037, ηp^2^ = 0.039) and the cognitive-affective score (*U* = 382.5, *z* = −2.407, *p* = 0.016, ηp^2^ = 0.055) on the BDI. There were no significant differences in self-reported depressive symptoms between the participants with a higher vs. lower degree of modulation on the other VEP components; C1, P1, and N1.

#### Associations Between Visual Evoked Potentials Modulation and Self-Reported Stress

A modest significant negative correlation using Spearman’s *rho* were found between averaged modulation of the P1-N1 component and the total score of PSS-10 [*r*_*s*_(109) = −0.265, *p* = 0.005]. A modest positive significant correlation was found between P1-N1 modulation and the perceived self-efficacy factor [*r*_*s*_(108) = 0.259, *p* = 0.007], and a moderate negative correlation was seen between P1-N1 modulation and the perceived helplessness factor [*r*_*s*_(109) = −0.222, *p* = 0.020]. Further, there was a significant correlation between averaged P1 modulation and the perceived self-efficacy factor of the PSS [*r*_*s*_(108) = 0.216, *p* = 0.025]. Only the associations between P1-N1 modulation and the total score of the PSS-10 as well as the perceived self-efficacy factor survived correction for multiple comparisons (*Bonferroni corrected* α = 0.0125).

A Mann-Whitney *U*-test showed significant differences between participants with high vs. low degree of modulation on the P1-N1 component both on total score (*U* = 411, *z* = −2.858, *p* = 0.004, ηp^2^ = 0.074) and both underlying factors; perceived helplessness (*U* = 444.5, *z* = −2.573, *p* = 0.010, ηp^2^ = 0.060) and perceived self-efficacy (*U* = 468, *z* = −2.335, *p* = 0.020, ηp^2^ = 0.050). In addition, there were significant differences between participants with high (*n* = 14) vs. low (*n* = 96) modulation on the P1 component on both total score (*U* = 433, *z* = −2.106, *p* = 0.035, ηp^2^ = 0.040) and the perceived helplessness factor on the PSS-10 (*U* = 433.5, *z* = −2.103, *p* = 0.035, ηp^2^ = 0.040). No significant differences were found between participants with high vs. low degree of modulation on the C1 and N1 component, respectively.

#### Associations Between Visual Evoked Potentials Modulation and Self-Reported Executive Function

**FIGURE 5:**
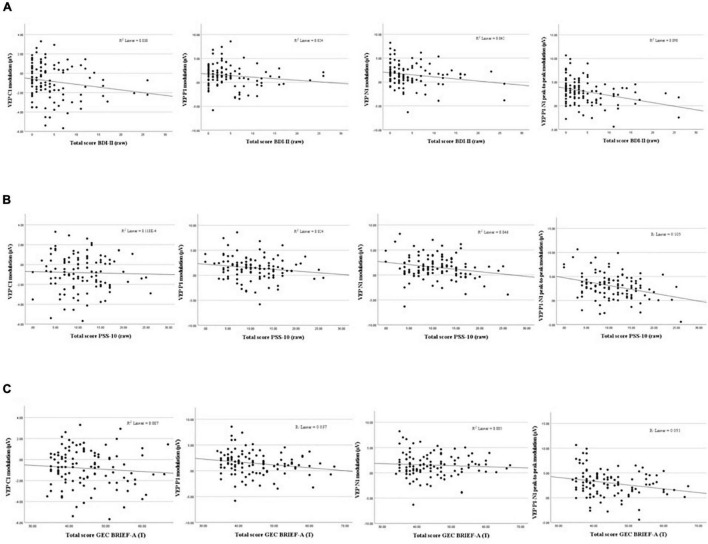
Scatterplots showing associations between VEP Plasticity (C1, P1, N1, and P1-N1) and self-reported depressive symptoms **(A)**, perceived stress **(B)**, and executive dysfunction **(C)**.

After correcting for multiple comparisons (*Bonferroni corrected* α = 0.0125), none of the correlations between composite scores and P1-N1 VEP plasticity reached statistical significance, though uncorrected the analyses showed significant associations: MI [*r*_*s*_(109) = −0.208, *p* = 0.030], BRI: [*r*_*s*_(109) = −0.236, *p* = 0.014], GEC: [*r*_*s*_(109) = −0.219, *p* = 0.022]. See Table 1 for correlation analyses between P1-N1 modulation across blocks and self-reported psychological distress, and Figure 5 for scatter plots illustrating correlation between VEP plasticity across components and self-reported psychological distress.

**TABLE 1:** Spearman correlation analyses (*N* = 109) between P1-N1 amplitude modulation of different post high frequency stimulation (HFS) time points and self-reported depressive symptoms (BDI-II), perceived stress (PSS-10), and executive function (BRIEF-A).

	P1N1 post1 (rho/p)	P1N1 post2 (rho/p)	P1N1 post3 (rho/p)	P1N1 post4 (rho/p)	P1N1 post1-4 (rho/p)
BDI-II total score	−0.147 *p* = *0.128*	−0.262** *p* = *0.006*	−0.251** *p* = *0.009*	−0.290** *p* = *0.002*	−0.319** *p* = *0.001*
BDI-II cognitive-affective	−131 *p* = *0.186*	−0.297** *p* = *0.002*	−0.288** *p* = *0.003*	−0.312** *p* = *0.001*	−0.340** *p* = < *0.001*
BDI-II somatic	−0.191 *p* = *0.052*	−0.246** *p* = *012*	−0.207* *p* = *0.035*	−0.258** *p* = *0.008*	−0.299** *p* = *0.002*
PSS-10 total score	−0.057 *p* = *559*	−0.265** *p* = *0.005*	−0.276** *p* = *0.004*	−0.215* *p* = *0.025*	−0.265** *p* = *0.005*
PSS-10 perceived self-efficacy	0.103 *p* = *289*	0.262** *p* = *0.006*	0.247** *p* = *0.010*	196* *p* = *0.043*	0.259** *p* = *007*
PSS-10 perceived helplessness	−0.053 *p* = *0.582*	−0.190* *p* = *0.048*	−0.231* *p* = *0.016*	−0.237* *p* = *0.013*	−0.222* *p* = *020*
BRIEF-A global composite	−0.051 *p* = *0.600*	−0.179 *p* = *0.063*	−0.240* *p* = *0.012*	−0.287** *p* = *0.002*	−0.223* *p* = *0.020*
BRIEF-A behavioral index	−0.055 *p* = *0.571*	−0.196* *p* = *0.041*	−0.227* *p* = −*017*	−0.255** *p* = *0.007*	−0.226* *p* = *0.018*
BRIEF-A metacognitive index	−0.063 *p* = *0.518*	−0.161 *p* = *0.095*	−0.214* *p* = *0.026*	−0.292** *p* = *0.002*	−0.208* *p* = *0.030*

**Correlation is significant at the 0.01 level (2-tailed).

*Correlation is significant at the 0.05 level (2-tailed).

*Correlations surviving correction for multiple comparisons in bold.*

A two independent samples Mann-Whitney *U*-test did not yield significant differences in any VEP components between participants with scores above the significant cut-off point as described in see section “Self-Report Questionnaires” on either of the composite scores; neither the MI, the BRI or the GEC.

**FIGURE 6:**
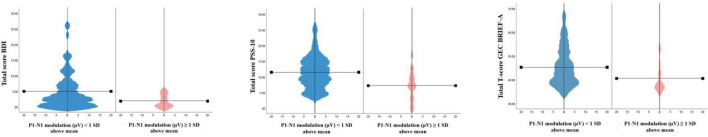
Violin plots showing differences between participants with higher (>1 SD above mean, *n* = 16) and (≤1 SD above mean, *n* = 94), respectively. P1-N1 modulation (μV) on depressive symptoms, perceived stress and executive dysfunction.

A Mann-Whitney *U*-test to examine differences between participants with higher vs. lower degree of modulation on the P1-N1 component did, however, show significant differences on both the metacognitive index (*U* = 431, *z* = −2.685, *p* = 0.007, ηp^2^ = 0.066), the BRI (*U* = 514, *z* = −1.973, *p* = 0.049, ηp^2^ = 0.035) and the global composite score (*U* = 442.5, *z* = −2.586, *p* = 0.010, ηp^2^ = 0.061). There were no significant differences on the BRIEF-A between participants with higher vs. lower modulation effect on either the C1, P1, or N1 components. See Figure 6 for violin plots illustrating the difference between participants with higher vs. lower modulation scores on the total score of BDI-II and PSS-10 and the GEC score on the BRIEF-A, respectively.

## Discussion

The present study found, in line with previous studies, a significant modulation effect following high-frequency visual stimulation, evident as VEP amplitude changes. The current results strengthen the notion that high-frequent visual stimulation is a viable, non-invasive probe into what has been labeled *LTP-like* synaptic plasticity in the visual cortex. More importantly, and representing a novel finding, the current study found significant moderate negative correlations between SRM visual synaptic plasticity and self-reported assessment of depressive symptoms and stress in a healthy population. There was no indication that different symptom clusters of depression contribute uniquely to the plasticity impairment, as the cognitive-affective factor of the BDI-II displayed only a marginally stronger correlation with SRM synaptic plasticity compared to the somatic factor of the BDI-II. Likewise, we found no apparent unique contributions from the latent factors of the PSS-10 on SRM visual synaptic plasticity, and neither survived *Bonferroni* correction. After correcting for multiple comparisons, none of the composite scores of the BRIEF-A indexing executive dysfunction correlated significantly with the modulation score indicating SRM synaptic plasticity, though they were trending toward significance.

Recent theoretical models seek to create a more comprehensive theory on neuroplasticity and depression by integrating different levels of analyses, linking molecular models of impaired plasticity with cognitive and behavioral symptoms of depressive disorders (Duman et al., 2017; Price and Duman, 2020). The neuroplasticity hypothesis of depression argues that an increase of glucocorticoids due to prolonged or traumatic stress act on multiple levels of neuronal function and behavior. Elevated levels of glucocorticoids will in turn disrupt mood-related circuitry involving the PFC and the hippocampus, brain regions implicated in cognitive functions such as attention, memory and cognitive flexibility (Price and Duman, 2020). It is well-documented that impairments in these cognitive domains are associated with depressive states (e.g., Landrø et al., 2001). Animal studies have demonstrated that exposing rodents to stress-inducing paradigms mimicking traumatic or chronic stress leads to neuronal atrophy and reduction of synapses in the PFC and hippocampus (Sapolshy, 2013). Consistent with what is seen in animal models, imaging studies show robust evidence of volume reduction in both cortical and limbic regions, including PFC and hippocampus, in depressed patients (Sheline et al., 1996; Pizzagalli and Roberts, 2021). The reduction in hippocampal volume seems to be correlated with the number of depressive episodes, and thus cumulate over time, indicating that greater functional impairment is associated with more severe depressive illness. Chronic stress has also been shown to reduce neurogenesis in the adult hippocampus, in contrast to pharmacological antidepressant treatments that seem to facilitate synaptic plasticity and increase neurogenesis (Castrén and Hen, 2013). However, presuming that the SRM-effect reflect LTP-like synaptic plasticity and knowing that LTP is present throughout the brain, there is a possibility that the modulation effect shown in the current and similar studies reflect a more general neural plasticity phenomenon not restricted to specific brain areas.

The current study found significant negative correlations between impaired neuroplasticity and self-reported symptoms of psychological distress in a population of healthy adults. This association can be seen as lending support to the neuroplasticity hypothesis of depression as well as previous research in clinical groups that has found impaired plasticity in individuals with uni- or bipolar depression (Normann et al., 2007; Elvsåshagen et al., 2012; Zak et al., 2018). The current results also support recent research providing evidence indicating that LTP-like synaptic plasticity is partially occluded in MDD, and restored in remission (Kuhn et al., 2016), as well as evidence indicating that LTP-like synaptic plasticity is negatively correlated with the severity of depression symptoms (Normann et al., 2007; Zak et al., 2018). These reports, in conjunction with the results of the current study, support the notion that symptoms of psychological distress exist on a continuum where more severely depressed individuals display a larger impairment in synaptic plasticity.

Depression presents as a heterogeneous illness where both genetic, epigenetic, endocrine and environmental risk factors may influence the onset, duration and severity of the disorder (Duman et al., 2017). Current clinical practice dictates that a diagnosis of depression relies on normative cut-off scores on a variety of assessment tools as well as a consensus around relevant clinical characteristics as stated by diagnostic manuals such as the ICD-10 and DSM-V (Goldman et al., 1991). The heterogeneous nature of depression elevates the importance of identifying possible underlying pathophysiological mechanisms that may aid in the understanding and treatment of depressive disorders. As both biological and environmental vulnerability factors may contribute to the development of depressive disorders, impairments in synaptic plasticity may be conceptualized as representing a possible biomarker of vulnerability to depressive symptomatology.

Developing a robust biomarker of sensory-induced LTP-like plasticity is of potential importance for clinical research and practice. Recent research by Jacob et al. (2021) demonstrated deficits in LTP-like VEP plasticity in a population of adolescents with psychosis risk syndrome (PRS) that later progressed to full-blown psychosis, relative to PRS individuals who did not progress (Jacob et al., 2021). Similarly, future studies should administer a VEP paradigm on a group of otherwise healthy adults with subclinical depression, and track which individuals subsequently progress to MDD. Identifying biomarkers of subclinical or prodromal symptoms of psychiatric illness can potentially guide intervention or treatment strategies and allow earlier intervention for these conditions. The current study indicates that even subclinical levels of depressive symptoms may lead to impairments in synaptic plasticity. To further explore this association, future studies should include participants with greater variability in depressive symptoms, ranging from an absence of symptoms to severe depressive symptoms, to strengthen the assumption that the magnitude of symptoms correspond with the impairment in LTP-like synaptic plasticity. More research is needed to further elucidate the associations between impairments and irregularities in synaptic function and plasticity and symptoms of psychological distress.

### Strengths and Limitations

A strength of the current study is the number of participants, which exceeds that of most comparable VEP studies. A limitation with the current study is that no formal diagnostic assessment of the participants was carried out. Hence, we cannot rule out that certain participants indeed suffered from undiagnosed ongoing psychiatric illness exceeding subclinical levels of psychological distress, despite the exclusion criteria stating no current or previous severe psychiatric disorders.

In addition, the current experimental design did not include administering a control stimulus to a subset of participants in addition to the high-frequent checkerboard stimulus. To ensure that the modulation effect was indeed induced by the high-frequent stimulation, a control group that was not exposed to the high-frequent stimulation could have been included. However, several studies have controlled for this, and found that modulation of VEP amplitudes post-stimulation only occurs after either prolonged (Normann et al., 2007) or high-frequent (Jacob et al., 2021) visual stimulation. To further ensure that the modulation effect reflects a synapse-specific enhancement similar to LTP rather than a more unspecific excitability induced by the high-frequency stimulation, the study design could also have included additional experimental factors exploring, e.g., spatial frequency (McNair et al., 2006), orientation (Ross et al., 2008), or eye-specific modulation (Teyler et al., 2005). As input-specificity is one of the hallmarks of LTP, this would strengthen our assumption that we are indeed inducing an LTP-like effect.

Despite considerable progress in the research on SRM synaptic plasticity as indexed by sensory stimulation in recent years, there is still considerable variability in the reported sensory-induced LTP-like effects *in vivo*, which needs further clarification. A lack of consensus on which characteristics of the stimuli elicits the most robust modulation effects, (e.g., high-frequent or prolonged stimulation, checkerboard vs. sinusoidal gratings), as well as what participant characteristics affect sensory plasticity, warrants further exploration of this phenomenon (see Sanders et al., 2018 for an extensive review).

## Conclusion

In summary, the current study found that visual high-frequent stimulation produces robust changes in amplitude modulation in healthy adults, as well as demonstrating moderate negative significant associations between VEP modulation and self-reported psychological distress. Considering the available body of evidence assessing LTP-like plasticity through stimulus-specific response modulation, it seems reasonable to consider this a phenomenon with a solid empirical foundation. In addition, the current results support the notion that LTP-like synaptic plasticity represents a potential biomarker of vulnerability for depressive symptomatology.

## Data Availability Statement

The raw data supporting the conclusions of this article will be made available by the authors, without undue reservation.

## Ethics Statement

The studies involving human participants were reviewed and approved by the Regional Ethics Committee, South-East Norway. ref. no: 2016/2003. The patients/participants provided their written informed consent to participate in this study.

## Author Contributions

SA, CH-H, TE, and TM designed the experiment. TR collected data. CH-H designed the MATLAB script and pre-processed the data. TR analyzed data and wrote the manuscript under supervision of SA and revisions from CH-H and TM. All authors contributed to the article and approved the submitted version.

## Conflict of Interest

The authors declare that the research was conducted in the absence of any commercial or financial relationships that could be construed as a potential conflict of interest.

## Publisher’s Note

All claims expressed in this article are solely those of the authors and do not necessarily represent those of their affiliated organizations, or those of the publisher, the editors and the reviewers. Any product that may be evaluated in this article, or claim that may be made by its manufacturer, is not guaranteed or endorsed by the publisher.
